# The Needs of To-Day

**Published:** 1911-06-18

**Authors:** 


### Hospital Sunday Number.

### The Needs of To-Day.

An arresting sentence in a chapter on "Hospital Sunday," taken from "Burdett's Hospitals and Charities" for 1911, may well give pause to every thoughtful reader. It is this. "The Hospital Sunday movement requires an apostle of the type of the prophets of old to re-create and vivify the spirit which the Hospital Sunday Movement formerly embodied."

If one doubts the truth of these words, reference to the figures representing the amounts contributed by congregations during the thirty-eight years the Fund has been in existence will prove that they are no exaggeration. While the number of contributing congregations has exactly doubled since 1873, the total amount collected has diminished. Why is this? Is the need for hospital accommodation less? Do the people no longer want hospital beds or are they so ably supported that they no longer require to appeal urgently for continued help? All these questions can be met by an unhesitating negative. Year by year hospitals are being used more and more, not only by the very poor, but increasingly by those of the middle classes who are unable to pay the heavy fees of expensive nursing homes, while requiring treatment only possible in a well-equipped hospital. Long lists of waiting patients keep every available bed continuously occupied, exchequer, accommodation, and nursing capacity often being equally overstrained to supply for every twenty what is really only sufficient for ten.

### Hospital Needs of To-day.

The needs of the hospitals to-day are more urgently patent than ever. Money will supply most of those needs, but the difficulty is to get people to recognise their personal responsibility in the matter. Sixpence or a shilling dropped perfunctorily into the offertory bag on Hospital Sunday will not release the donor from this responsibility. Our common mortality compels from the healthy a vital interest in the care of the sick. Religious societies may not appeal to all. Purses may be closed against sects and creeds; but in the hospitals all meet on the ground of suffering humanity, and none know whose turn it may be next to lie bound in the chain of sickness.

### The Most Vital Need.

The greatest need of almost all hospitals to-day is room for expansion in order to keep pace with the requirements of rapidly growing districts, the constant improvements in sanitary science, and the continued advance of preventive treatment as well as surgical knowledge. Inadequate buildings, overcrowded wards, lack of labour-saving appliances, an insufficient nursing staff, perhaps indifferently fed and badly housed, all help to stultify and cramp the best efforts of physicians and surgeons in their perennial fight against disease and death. The best of everything cannot be afforded owing to lack of funds, and praiseworthy shift is made with the second best, the human element usually rising nobly to the occasion of extra strain; but the results cannot be as good as they might be, and the best workers get worn out before their time. Yet in spite of the obvious need for expansion, there are some who refuse to help on the ground that the hospitals are always begging for more and are never satisfied. They forget that all healthy life is always growing and ever in need of more room, more support. Surely they can never have considered the matter, or they would not wish that the hospitals should stand still and not seek to enlarge their borders.

### Personal Interest.

There need be no lack of knowledge on the part of the public about hospitals to-day. Scarcely any other kind of work is so much written about, commented upon, and criticised in the daily Press and current magazine literature; but bare knowledge of facts alone will not arouse interest enough to loosen the purse-strings unless the personal element enters into it. More co-operation between the hospitals and the general public is badly needed; co-operation with churches and chapels, irrespective of creed; with schools, factories, places of business, railway companies; co-operation between the official workers inside the hospitals, and individual members of boards, committees, and councils.

### Hospitals as Harbours of Refuge.

There are many annual subscribers of a guinea who would willingly make it ten if they would but personally visit the hospital subscribed to, and become really acquainted with what goes on within its walls. The histories of some of those quiet figures in the neat beds, told in their own words, would rouse their dormant sympathies by their simple human interest in a way that no sermon, however eloquent, and no study of statistics could ever do. They would see for themselves that work-worn, middle-aged woman, arrayed for the first time since her marriage, twenty years ago, in a complete suit of new clothing supplied by the Samaritan Fund of the hospital, and would hear her saying how she is going to a convalescent home after a severe operation, adding quietly, in a matter-of-fact way, that she has "never had a holiday in her life before." They would notice the small boy whose clothing has been burnt off him in a serious accident, also stiffly proud in a new suit, but they would observe that his boots were old and tied up with string. Alas! the hospital resources have given out at this point, for new boots are not often given, or the money to buy them, and they are expensive items for the poor And they would doubtless reflect that there were many hitherto unthought-of ways of helping the hospitals, and that boots, clothing, and convalescent home letters would greatly relieve the strain on hospital beds, giving the patients themselves also far better chance of re-equipment for the battle of life. Here, too, they would see the flotsam and jetsam of our everyday life; the wastrel, the harlot, side by side with the innocent, the pure, all invested with the nameless dignity of suffering. Even the silent, bandaged form in the screened bed, watched by police night and day, though he has tried unsuccessfully, the " coward's exit " from the " dolour of the fight," is not rejected by the hospitals. Truly they are cities of refuge for those who are " bankrupt of hope."

Shall the spirit of co-operation, which alone can adequately serve our sick brothers and sisters—this bond of union between the hospital worker and outside helper, die out? The Hospital Sunday movement, the very embodiment of that spirit of unity, only needs fanning once more into vigorous life to reanimate whole congregations with a freshness of interest born of a common cause appealing to all alike. Workers are needed by the hospitals, not figure-heads, or mere fair-weather friends, but those possessed of an earnestness which will not be daunted, a courage that nothing will quench, men and women whose hearts God has touched, who are willing to give money, time, and work to the sacred service of the sick.

